# Creative, yet not unique? Paranormal belief, but not self-rated creative ideation behavior is associated with a higher propensity to perceive unique meanings in randomness

**DOI:** 10.1016/j.heliyon.2022.e09269

**Published:** 2022-04-12

**Authors:** Christian Rominger, Andreas Fink, Corinna M. Perchtold-Stefan, Günter Schulter, Elisabeth M. Weiss, Ilona Papousek

**Affiliations:** a Institute of Psychology, University of Graz, Austria; b Institute of Psychology, University of Innsbruck, Austria

**Keywords:** Pareidolias, Patternicity, Inkblot, Rorschach, Meaningful coincidences

## Abstract

Apophenia, patternicity, and the experience of meaningful coincidences describe the propensity to perceive meaning in random arrangements, which is known to be linked with paranormal beliefs. Additionally, this trait of combining unrelated elements to create new meanings suggests an association with creativity. However, studies indicating a relationship between creativity and apophenia are scarce. To gain empirical evidence, the present study (n = 77) assessed the propensity to experience meaningful patterns in random arrangements by means of a questionnaire (coincidence questionnaire) and a behavioral measure. The applied figural association task allows to reliably differentiate between the perception of idiosyncratic/unique and intersubjective meaningful/non-unique patterns. Self-rated creative ideation behavior and paranormal beliefs were positively associated with the subjectively rated frequency of meaningful coincidences. Furthermore, participants high in both creative ideation behavior and paranormal beliefs perceived a higher number of non-unique meanings in the figural association task. Yet, participants high in paranormal beliefs additionally perceived a higher number of unique meanings. This divergence in findings suggests that creative ideation behavior and paranormal belief are associated with the perception of partly different meanings in random arrangements. In paranormal believers, this pattern of findings may indicate a lower threshold to detect meaning in meaninglessness, leading to more idiosyncratic/unique perceptions. Altogether, slight reductions of this threshold to detect meaningfulness may increase a persons’ creativity; however, excessive pattern recognition may facilitate paranormal beliefs.

## 1 Introduction

There is a long history of scientific investigation of the link between creativity and madness on a clinical as well as subclinical level (for overviews see Abraham, 2015; DeYoung et al., 2012; Fink et al., 2014a; Kaufman and Paul, 2014; Rominger et al., 2021). This body of research indicates that specific aspects of the non-clinical positive schizotypal trait (e.g., magical thinking, allusive thinking, unusual perceptual experiences; see e.g., Raine, 1991) are positively related with creativity while aspects of negative schizotypy (e.g., diminished functioning, flattened affect; see e.g., Chun et al., 2019) show negative associations with creativity (Acar and Sen, 2013; Baas et al., 2016; Byron and Khazanchi, 2011). As a result, predominantly non-clinical personality traits should be linked with creativity (Fink et al., 2014a).

In this study we investigated two personality traits that have been related to positive aspects of schizotypy, but that have been largely neglected in creativity research: paranormal beliefs and the propensity to experience meaningful coincidences (Hergovich et al., 2008; Partos et al., 2016; Rominger et al., 2011; Stumm and Scott, 2019; Thalbourne and Delin, 1994). Paranormal beliefs, defined as beliefs which are currently unexplained by science (Irwin, 1999), were found to be linked with creative personality (Stumm and Scott, 2019; Thalbourne and Delin, 1994) and the detection of meanings in random arrangements, such as seeing faces in everyday objects (Blackmore, 1994; Brugger et al., 1993; Krummenacher et al., 2010; Riekki et al., 2013; Rominger et al., 2011; Sannwald, 1962; but see Farias et al., 2005). The propensity to perceive meaning in meaningless noise is called apophenia, patternicity, pareidolias, or the experience of meaningful coincidences (in a predominantly temporal sense; Beitman, 2009; Brugger, 2001; DeYoung et al., 2012; Diaconis and Mosteller, 1989; Partos et al., 2016; Rominger et al., 2018; Shermer, 2008). Being able to perceive what *remains hidden from the view of others* was suggested to be an important feature of creative modes of thinking (cf. Carson et al., 2003; Brugger et al., 1993; Brugger and Regard, 1995; Cheng et al., 2016; DeYoung et al., 2012; Griffiths and Tenenbaum, 2007; Kazemzadeh, 2012; Mohr et al., 2001; Wiseman et al., 2011). For this reason, it may be of particular interest to empirically investigate the association between the experience of meaningful patterns, paranormal beliefs, and creative ideation behavior.

The propensity to perceive meaningful patterns in random arrangements can be measured by behavioral tests and questionnaires. Behavioral measures allow a direct observation of apophenia, by asking participants to indicate when they perceive meaning in random arrangements of dots, or inkblots (Brugger et al., 1993; Brugger and Regard, 1995; Farias et al., 2005; Rominger et al., 2018; for further behavioral methods see Blain et al., 2020; Fyfe et al., 2008; Partos et al., 2016). This approach allows to assess the content of perceived patterns and the application of frequency analyses. With these more detailed analyses, a differentiation between unique answers (only perceived by one person) and non-unique answers, which are perceived by more than one person and therefore are intersubjectively meaningful, is possible (Rominger et al., 2018). In contrast to the behavioral approach, which is restricted to assessment conditions in the laboratory, questionnaires allow the assessment of the experience of meaningful patterns in everyday life. The coincidence questionnaire measures the frequency of meaningful coincidences (Bressan, 2002), which are surprising concurrences of events, with no apparent causal connection, such as receiving an incoming phone call from a friend you have not met for a long time, exactly at the moment when you are thinking about this very friend (cf. Diaconis and Mosteller, 1989, p. 853). In a recent study, the self-rated propensity to experience meaningful coincidences was significantly associated with an increased number of perceived patterns in a behavioral association test of randomly arranged figural stimuli, which were intersubjectively meaningful (i.e., non-unique; Rominger et al., 2018). Most interestingly, in this study, the propensity to perceive meaningful coincidences and the number of intersubjectively meaningful objects (non-unique associations) were both associated with similar neurophysiological deviations in early attentional processes. This indicates that both findings from a questionnaire and a behavioral measure converge in similar neurophysiological processes, arguing for a shared underlying neuronal mechanism of the propensity to perceive meaning in randomness (for further structural and functional brain properties of perceivers see Rominger et al., 2019; Unger et al., 2021).

Although many authors have suggested a relationship between apophenia and creativity (Brugger, 2001; DeYoung et al., 2012; Rominger et al., 2011), the empirical evidence is still limited, since to date, only a few studies are available on this topic. Diana et al. (2020) showed that divergent thinking performance (i.e., the production of as many original ideas as possible) is associated with the propensity to detect various meanings in pictures of natural landscapes, which indicates that creativity and apophenia might share some cognitive functions. In accordance with this, Rominger et al. (2017) found that participants with higher creative potential as indexed by better performance in divergent thinking tasks also perceived a higher number of patterns in randomly arranged figural stimuli. However, this work did not differentiate between the number of unique and non-unique answers. While unique answers indicate the perception of idiosyncratic meaning and a lower threshold to detect meaning in meaninglessness, non-unique answers are intersubjectively meaningful and are specifically associated with the perception of meaningful coincidences (Rominger et al., 2018). Since this ongoing field of research still needs further empirical evidence, the current study aimed at investigating potential links between creative ideation behavior, paranormal beliefs, and the propensity to perceive meaning in meaninglessness. We assessed apophenia by means of a questionnaire and a behavioral figural association test, which allows a more detailed, in-depth assessment of the number of meaningfully perceived patterns by differentiating between unique and non-unique perceptions (Asari et al., 2008, 2010; Rominger et al., 2018).

Based on available literature, we hypothesized that self-rated creative ideation behavior and paranormal beliefs would be positively linked with the propensity to perceive meaningful coincidences, both when assessed by self-report in the coincidence questionnaire and behaviorally by the number of intersubjectively meaningful patterns in an inkblot test (Rominger et al., 2018). We further examined whether the perception of unique/idiosyncratic meanings show associations with creative ideation behavior and paranormal beliefs.

## 2 Methods

### 2.1 Participants

An a-priori calculated power analysis using the software GPower 3.1 (Faul et al., 2009) indicated that a sample size of 77 participants was required to detect a medium effect (*f*^*2*^ = .15) using a power of .80 and a type I error probability of 5% (three predictors). Therefore, eighty participants were sampled in this correlational design study. All participants had to be right-handed (indicated by a standardized handedness test, HDT; Steingrüber, 2010) and had to report a negative history of any psychiatric or neurological illness. Based on these criteria, three participants had to be excluded because they reported a clinically relevant depression. The final sample consisted of 77 participants (42 women and 35 men) with an age range between 18 and 43 years (*M* = 23.55, *SD* = 4.87), which allowed the detection of medium to large effects observed in the literature (Rominger et al., 2017). Ethics approval was granted by the Ethics Committee of the University of Graz (reference number: GZ. 39/93/63 ex 2020/21). Informed consent was obtained from all participants.

### 2.2 Figural association task: A behavioral measure of apophenia and the propensity to experience meaningful patterns in randomly arranged stimuli

In this task 33 published inkblots (Drey Fuchs, 1958; Rorschach, 1949; Zulliger, 1946, 1951) were randomly presented (10 s) for the behavioral assessment of the propensity to perceive meaningful patterns in randomly arranged stimuli (Rominger et al., 2018). It is important to note that the figural association task strives to measure the propensity to perceive meaning in random stimuli by naming the first association which comes into participants’ mind. Participants were instructed to name the object they perceived, if any, when looking at the figural stimuli, and then described the meaning of the inkblot with one noun by writing their answer on a sheet of paper. Subsequently, the presentation of a new stimulus was indicated by an auditory signal.

Following former work on the coding of word association tests, perceived patterns were classified as unique (perceived by only one person see, e.g., Asari et al., 2010; Duchêne et al., 1998; Gianotti et al., 2001; Rominger et al., 2011; Rominger et al., 2017). The number of perceived unique patterns (*M* = 8.90, *SD* = 5.22) indicate idiosyncratic meanings, which are not experienced by others. In accordance with Rominger et al. (2018), non-unique and intersubjectively meaningful answers, which were perceived by more than one participant, served as a measure of the propensity to perceive meaningful patterns in randomly arranged stimuli (*M* = 14.39, *SD* = 4.02). The categorization of perceived patterns into unique and non-unique (i.e., idiosyncratic and intersubjectively meaningful) was based on a sample of 206 participants gathered in pilot studies and from published studies (Rominger et al., 2017, 2018). The split-half reliability (Spearman-Brown coefficient) was good for idiosyncratic/unique patterns (*r* = .84) and for the number of intersubjectively meaningful patterns (*r* = .76). The number of idiosyncratic patterns was unrelated to the number of intersubjectively meaningful patterns (*r* = -.01, *p* = .919), indicating two largely independent measures of pattern recognition in figural stimuli, which strongly confirms their differentiation in the present study.

### 2.3 Questionnaires

#### 2.3.1 Coincidence questionnaire: The propensity to perceive meaningful patterns in coincident events

A German translation of the Coincidence Questionnaire was administered (Bressan, 2002; Rominger et al., 2011). This questionnaire assesses how frequently participants experience several categories of "meaningful" coincidences in their everyday life (e.g., "spontaneous associations" like thinking of someone and running unexpectedly into that person soon afterwards; “perception of something distant in time” like having a dream that then comes true). The scale has 7 items, which are rated on a 5-point Likert scale from "never" to "very often". The sum of all items indicates the propensity to perceive meaningful coincidences (*M* = 17.69, *SD* = 3.95). In this study, the Cronbach's alpha was acceptable but moderate with α = .64.

#### 2.3.2 Ideational Behavior Scale: Self-assessment of creative ideation behavior

We assessed creative ideation behavior by means of a German version of Runco's Ideational Behavior Scale (RIBS; Runco et al., 2001; see e.g., Benedek et al., 2012; Edl et al., 2014; Rominger et al., 2022). The RIBS includes 17 positively coded statements, like “I come up with an idea or solution other people have never thought of”. The scale was constructed as a criterion measure of creative idea production and reflects creative ideation skills (Runco et al., 2014). Therefore, the RIBS shows associations with divergent thinking performance in various tasks assessing participants' creative potential to solve open problems (Benedek et al., 2012; Fink et al., 2014b; Plucker et al., 2006; for an overview see Runco et al., 2014). Participants responded to the items on a scale ranging from 1 (never) to 5 (very often; *M* = 66.45, *SD* = 14.12; α = .91).

#### 2.3.3 The revised Paranormal Belief Scale (rPBS)

A German translation of the revised Paranormal Belief Scale (Tobacyk, 2004) was used to measure the belief in paranormal phenomena (Rominger et al., 2011). The questionnaire consists of 26 statements (e.g., “Some people have an unexplained ability to predict the future”; 5-point Likert scale; *M* = 45.61, *SD* = 13.25) and showed a good internal consistency of α = .89 in the present study.

### 2.4 Statistical analyses

To determine how specific aspects of the propensity for experiencing meaning in random arrangements are linked to paranormal beliefs and creative ideation behavior, multiple regression analyses were run. The two behavioral scores from the figural association test (number of unique and non-unique patterns) were simultaneously entered as predictors. The resulting semi-partial correlations allowed to determine whether paranormal belief and creative ideation behavior were uniquely related to specific aspects of perceived meaning. We included gender as an additional predictor to control for potential gender differences in paranormal beliefs (Aarnio and Lindeman, 2005), creative ideation behavior (Batey et al., 2010), as well as the propensity to experience meaningful coincidences (Bressan, 2002). Furthermore, two analogous regression analyses were calculated with self-report in the coincidence questionnaire and gender as predictors of paranormal beliefs and self-rated creative ideation behavior. The significance level was set to *p* < .05 (two-tailed). All analyses were calculated with SPSS 25.

## 3 Results

### 3.1 Perceived meanings in the figural association task

The standard multiple regression analysis with self-reported creative ideation behavior as dependent variable was significant, *F*(3,73) = 8.39, *p <* .001. Independent of gender the number of intersubjectively meaningful/non-unique patterns (*sr* = .28, *p* = .007), but not the number of idiosyncratic/unique patterns (*sr* = -.03, *p* = .778) predicted creative ideation behavior (see Table 1). Furthermore, gender significantly predicted creative ideation behavior (*sr* = -.44, *p* < .001), as men (*M* = 73.20, *SD* = 13.41) reported higher creative ideation behavior than women (*M* = 60.83, *SD* = 13.46).

**Table 1:** Creative ideation behavior and behavioral measures of apophenia

	R^2^	r	p	sr	p
Gender	.26	-.42	<.001	-.44	<.001
Idiosyncratic/unique patterns		.05	.679	-.03	.778
Intersubjectively meaningful patterns		.25	.030	.28	.007

Note. R^2^ = proportions of variance explained by the model in total, r = Pearson correlation; sr = semipartial correlation.

In contrast to creative ideation behavior, paranormal belief was predicted by all three variables, *F*(3,73) = 7.66, *p <* .001 (see Table 2). The number of intersubjectively meaningful patterns (s*r* = .27, *p* = .010), the number of idiosyncratic/unique patterns (s*r* = .29, *p* = .006), and gender were significant (*sr* = .32, *p* = .003). Men showed a lower paranormal belief score (*M* = 41.43, *SD* = 11.43) in contrast to women (*M* = 49.10, *SD* = 13.77).

**Table 2:** Paranormal beliefs and behavioral measures of apophenia

	R^2^	r	p	sr	p
Gender	.24	.29	.010	.32	.003
Idiosyncratic/unique patterns		.23	.043	.29	.006
Intersubjectively meaningful patterns		.29	.010	.27	.010

Note. R^2^ = proportions of variance explained by the model in total, r = Pearson correlation; sr = semipartial correlation.

### 3.2 Meaningful coincidences in everyday life

Self-rated creative ideation behavior was positively associated with the propensity to perceive meaningful coincidences in daily life independent from gender (*sr* = .24, *p* = .022; *F*(2,74) = 11.31, *p* < .001; *R*^*2*^ *=* .23). Similarly, participants with higher paranormal belief reported a higher propensity to experience meaningful coincidences (*sr* = .26, *p* = .018; *F*(2,74) = 6.59, *p =* .002; *R*^*2*^ *=* .15). Similar to the former regression analyses, gender was a significant predictor for creative ideation behavior (*sr* = -.43, *p <* .001) and paranormal beliefs (*sr* = .28, *p =* .012).

As expected, the propensity to experience meaningful coincidences was associated with the number of intersubjectively meaningful/non-unique patterns (*r* = .32, *p* = .004) and was not significantly associated with the number of idiosyncratic/unique patterns (*r* = .15, *p* = .188). Self-rated creative ideation behavior was not correlated with paranormal belief (*r* = -.07, *p* = .524).

## 4 Discussion

The present study investigated the association between self-rated creative ideation behavior and paranormal belief with the propensity to perceive meaning in randomness by means of a behavioral measure (i.e., figural association task) and a self-report questionnaire (i.e., coincidence questionnaire). Participants reporting a greater amount of creative ideation behavior generally perceived a higher number of intersubjectively meaningful (and non-unique) patterns in inkblots and additionally indicated a higher frequency of experiencing meaningful coincidences in their everyday life. Since Runco's Ideational Behavior Scale is a valid indicator of the creative potential of a person (Runco et al., 2014), our results corroborate a positive relationship between creative ideation performance and the propensity to perceive meaning in random arrangements (Rominger et al., 2017) and to see patterns in new ways (Wiseman et al., 2011). Furthermore, our finding is in accordance with Diana et al. (2020), who showed an association between participants' creative ideation performance and their skill in producing as many divergent illusory perception patterns as possible when looking at photographs of clouds and stones. But what makes these observations relevant for creativity?

It is plausible that the association between self-rated creative ideation behavior and the number of intersubjectively meaningful patterns may reflect aspects of participants’ originality - that is, an increased prevalence to transform random arrangements into meaningful patterns (Rominger et al., 2017). This interpretation is strengthened by studies, which reported psychometrically assessed originality negatively associated with the probability to come up with a common figural associations (Rominger et al., 2017) and negatively with the perceived semantic distance between words (Rossmann and Fink, 2010). These authors concluded that more creative people might have the skill to connect semantically unrelated elements to form new combinations and build new connections between stimuli. Furthermore, the predominant production of more intersubjectively meaningful patterns captures another important aspect of creativity: the production of appropriate and useful ideas when confronted with an open-ended problem (Runco and Jaeger, 2012). A certain level of intersubjectivity seems important in order for others to estimate the creative value of an idea and its appropriateness.

In accordance with the findings of self-rated creative ideation behavior, paranormal belief was positively linked with the number of intersubjectively meaningful patterns and with the propensity to perceive meaningful coincidences in daily life. This finding is in line with a wide variety of empirical research conducted during the last decades, which suggested that believers and non-believers differ in their imaginative behavior (Stumm and Scott, 2019), their perception of causality (Torres et al., 2020), and their cognitive and perceptual styles associated with apophenia (Blackmore, 1994; Blackmore and Troscianko, 1985; Brugger et al., 1993; Fyfe et al., 2008; Riekki et al., 2013; Rominger et al., 2011, 2019). Matching the current findings, Sannwald (1962) reported that people, who believed to have experienced paranormal phenomena gave more answers during an inkblot test compared to controls. However, a more in-depth analysis revealed that creative ideation behavior and paranormal beliefs showed different associations with the perception of meaning in random arrangements. In contrast to creative ideation behavior, paranormal belief correlated with an increased perception of idiosyncratic/unique meanings. This may indicate that paranormal believers have a reduced threshold for the detection of meaning in meaninglessness noise (Brugger and Graves, 1997; Partos et al., 2016; Riekki et al., 2013). In line with this, a study reported a lower probability to perceive common patterns in people with higher positive schizotypy (Rominger et al., 2017). Similarly, Sannwald (1962) reported less stereotypic answers of participants with paranormal experiences. Furthermore, more uncommon word associations in paranormal believers were reported (Gianotti et al., 2001) and Mohr et al. (2001) showed that people high in magical ideation considered unrelated words as more closely associated than their counterparts. In a similar context, authors suggested that increased associative processing in paranormal believers might be linked with right hemispheric functions (Pizzagalli et al., 2001). Interestingly, neurophysiological studies indicated that the perception of unique meanings in inkblots is underpinned by a specific activation of right temporal areas (Asari et al., 2008). This, in combination with the present findings is in accordance with studies suggesting deviations of right hemispheric activation as a neurophysiological correlate of paranormal beliefs (Brugger et al., 1993; Brugger and Regard, 1995; Leonhard and Brugger, 1998; Rominger et al., 2014). Taken together, this divergence of findings between self-rated creative ideation behavior and paranormal belief argues for the assumption that a little reduction of the threshold to detect meaning might make a person more creative in problem solving but excessive pattern recognition (associated with unique patterns) rather facilitates paranormal beliefs.

This conclusion is based on a reliable and valid breakdown of perceived meanings in random arrangements into idiosyncratic/unique and intersubjectively meaningful/non-unique patterns. This differentiation in combination with the application of a subjective assessment of meaningful coincidences constitute clear strengths of this study. When interpreting our findings, it should be noted that (1) reliability indices of both behavioral measures of perceived meaning in randomly arranged stimuli were good, (2) non-unique and unique meanings constitute two independent measures of the figural association task, and (3) the number of intersubjectively meaningful patterns is a valid behavioral measure of meaningful coincidences and apophenia (for a neurophysiological validation see Rominger et al., 2018).

But why are non-unique perceptions associated with apophenia and meaningful coincidences and not the number of perceived unique and idiosyncratic meanings? One potential answer is that assembling random elements into intersubjective meanings is at the core of apophenia and patternicity, such as seeing a man in the moon, perceiving human figures in nature, human faces on toasted bread, and hearing voices in random sounds (Merckelbach and van de Ven, 2001; Riekki et al., 2013; Shermer, 2008). Partos et al. (2016) reported that participants high in positive schizotypy predominantly experienced stereotypies such as human faces, people, and animals when presented with noisy visual information. Similar findings were also reported for patients with dementia suffering from hallucinations (i.e., Lewy Body Dementia; Uchiyama et al., 2012) when perceiving meaning in inkblots (see also Asari et al., 2008; for a more detailed discussion see Diana et al., 2020).

The present study is not without limitations. Contrary to our assumptions, paranormal belief was not significantly associated with creative ideation behavior (Stumm and Scott, 2019; Thalbourne and Delin, 1994), which may be attributed to the nature of our sample: We investigated young students, who naturally show reduced variance of paranormal beliefs and creative ideation behavior. However, the observed gender effects were nicely in accordance with literature, showing higher paranormal beliefs in women and higher creative ideation behavior in men (Aarnio and Lindeman, 2005; Batey et al., 2010). Additionally, creative ideation behavior and paranormal beliefs were associated with apophenia (independently from gender), which itself was reliably assessed via a behavioral test and a self-rated questionnaire. In accordance with previous work, the present research adds evidence to the notion that apophenia, patternicity, and the perception of meaningful coincidences are associated with aspects of creativity (Diana et al., 2020).

Following from this, future studies might apply a broader multi-measurement approach to assess creativity (see e.g., Agnoli et al., 2016). This would allow to investigate if creative ideation performance and creative achievements would show a similar pattern of associations with the number of perceived non-unique meanings, as we found for creative ideation behavior. Specifically, creative ideation performance measures would allow to evaluate if originality, fluency, or the flexibility component of creativity add to the association with apophenia (Diana et al., 2020). Additionally, study results should be replicated with more ecologically valid assessments of creativity and the experience of meaningful coincidences (e.g., by applying daily diary methods). In this regard, ecological momentary assessments (EMA; Shiffman et al., 2008) could measure these (subjective) phenomena at the very moment of occurrence, in ever changing situations of people's everyday life (Fahrenberg et al., 2007). Although EMA is already in use in creativity research (e.g., Benedek et al., 2017; Conner et al., 2018; Karwowski et al., 2017; Karwowski et al., 2021; for overviews see Cotter and Silvia, 2019; Rominger et al., in press), to the best of our knowledge, it has not been applied to assess the experience of meaningful coincidences to date.

## 5 Conclusion

Taken together, apophenia and the propensity to experience meaningful coincidences, traits of people to combine unrelated elements into new meanings, are interesting phenomena well suited to investigate the association with creativity, creative ideation behavior, and creativity-relevant personality traits such as paranormal beliefs, positive schizotypy, psychoticism, and openness in healthy people (Blain et al., 2020). For the very first time, this study reported an association between the perception of meaningful coincidences and creativity (for pareidolias see Diana et al., 2020). More creative people perceived more non-unique meanings in random arrangements as compared to less creative people. This was similar for believers in the paranormal. However, in contrast to creative people, believers additionally reported more idiosyncratic and unique meanings in random arrangements. Therefore, investigating meaningful coincidences and pattern recognition constitutes a valuable approach for parsing associations with creativity, paranormal beliefs, and madness on a subclinical level (Abraham, 2015; Fink et al., 2014a).

## Declarations

### Author contribution statement

Christian Rominger; Ilona Papousek: Conceived and designed the experiments; Analyzed and interpreted the data; Contributed reagents, materials, analysis tools or data; Wrote the paper.

Andreas Fink; Günter Schulter; Elisabeth M. Weiss: Contributed reagents, materials, analysis tools or data; Wrote the paper.

Corinna M. Perchtold-Stefan: Analyzed and interpreted the data; Wrote the paper.

### Funding statement

This research did not receive any specific grant from funding agencies in the public, commercial, or not-for-profit sectors.

### Data availability statement

Data will be made available on request.

### Declaration of interests statement

The authors declare no conflict of interest.

### Additional information

No additional information is available for this paper.
